# Federated learning and differential privacy for medical image analysis

**DOI:** 10.1038/s41598-022-05539-7

**Published:** 2022-02-04

**Authors:** Mohammed Adnan, Shivam Kalra, Jesse C. Cresswell, Graham W. Taylor, Hamid R. Tizhoosh

**Affiliations:** 1 Kimia Lab, University of Waterloo, Waterloo, Canada; 2 Vector Institute, MaRS Discovery District, Toronto, Canada; 3 Layer 6 AI, MaRS Discovery District, Toronto, Canada; 4 University of Guelph, Guelph, ON Canada; 5 Artificial Intelligence and Informatics, Mayo Clinic, Rochester, MN USA

**Keywords:** Cancer imaging, Biomedical engineering, Pathology

## Abstract

The artificial intelligence revolution has been spurred forward by the availability of large-scale datasets. In contrast, the paucity of large-scale medical datasets hinders the application of machine learning in healthcare. The lack of publicly available multi-centric and diverse datasets mainly stems from confidentiality and privacy concerns around sharing medical data. To demonstrate a feasible path forward in medical image imaging, we conduct a case study of applying a differentially private federated learning framework for analysis of histopathology images, the largest and perhaps most complex medical images. We study the effects of IID and non-IID distributions along with the number of healthcare providers, i.e., hospitals and clinics, and the individual dataset sizes, using The Cancer Genome Atlas (TCGA) dataset, a public repository, to simulate a distributed environment. We empirically compare the performance of private, distributed training to conventional training and demonstrate that distributed training can achieve similar performance with strong privacy guarantees. We also study the effect of different source domains for histopathology images by evaluating the performance using external validation. Our work indicates that differentially private federated learning is a viable and reliable framework for the collaborative development of machine learning models in medical image analysis.

## Introduction

Deep neural networks have achieved and established state-of-the-art results in many domains. However, deep learning models are data-intensive, i.e., they often require millions of training examples to learn effectively. Medical images may contain confidential and sensitive information about patients that often cannot be shared outside the institutions of their origin, especially when complete de-identification cannot be guaranteed. The European General Data Protection Regulation (GDPR) and the United States Health Insurance Portability and Accountability Act (HIPAA) enforce guidelines and regulations for storing and exchanging personally identifiable data and health data. Ethical guidelines also encourage respecting privacy, that is, the ability to retain complete control and secrecy about one’s personal information^1^. As a result, large archives of medical data from various consortia remain widely untapped sources of information. For instance, histopathology images cannot be collected and shared in large quantities due to the aforementioned regulations, as well as due to data size constraints given their high resolution and gigapixel nature. Without sufficient and diverse datasets, deep models trained on histopathology images from one hospital may fail to generalize well on data from a different hospital (out-of-distribution)^2^. The existence of bias or the lack of diversity in images from a single institution brings about the need for a collaborative approach which does not require data centralization. One way to overcome this problem is by collaborative data sharing (CDS) or federated learning among different hospitals^3^.

In this paper, we explore federated learning (FL) as a collaborative learning paradigm, in which models can be trained across several institutions without explicitly sharing patient data. We study the impact of data distribution on the performance of FL, i.e., when hospitals have more or less data, and IID or non-IID data. We also show that using federated learning with additional privacy preservation techniques can improve the performance of histopathology image analysis compared to training without collaboration and quantitatively measure the privacy using Rényi Differential Privacy Accountant^4^. We discuss its benefits, drawbacks, potential weaknesses, as well as technical implementation considerations. Finally, we use lung cancer images from The Cancer Genome Atlas (TCGA) dataset^5^ to construct a simulated environment of several institutions to validate our approach.

### Federated learning (FL)

Federated learning algorithms learn from decentralized data distributed across various client devices, in contrast to conventional learning algorithms. In most examples of FL, there is a *centralized server* which facilitates training a shared model and addresses critical issues such as data privacy, security, access rights, and heterogeneity^6^. In FL, every client locally trains a copy of the centralized model, represented by the model weights *ω*, and reports its updates back to the server for aggregation across clients, without disclosing local private data. Mathematically, FL can be formulated as:

1 $$\mathop {\min }\limits_{\omega \in R} f\left( \omega \right)\quad {\text{with}}\quad f\left( \omega \right) = \frac{1}{n}\sum\limits_{i = 1}^{n} {fi\left( \omega \right)} ,$$

where *f* (*ω*) represents the total loss function over *n* clients, and *f*_*i*_(*ω*) represents the loss function with respect to client *i*’s local data. The objective is to find weights *ω* that minimize the overall loss. McMahan et al.^6^ introduced federated averaging, or *FedAvg* (Algorithm 1), in which each client receives the current model *ω*_*t*_ from the server, and computes ∇ *f*_*i*_(*ω*_*t*_), the average gradient of the loss function over its local data. The gradients are used to update each client’s model weights using stochastic gradient descent (SGD) as $\omega_{t + 1}^{i} \leftarrow \omega_{t} - \eta \nabla f_{i} (\omega_{t} )$ according to the learning rate *η*. Next, the central server receives the updated weights $\omega_{{t + {1}}}^{i} \leftarrow \omega \sum\limits_{i = 1}^{n} {\frac{{n_{i} }}{n}\omega_{{t + {1}}}^{i} }$, where *n* is *t *+ 1 from all participating clients and averages them to update the central model, *t* + 1* ← i* = 1 *n* the number of data points used by client *i*. To reduce communication costs, several local steps of SGD can be taken before communication and aggregation, however, this affects the convergence properties of FedAvg^7^.

Other methods for FL have also been proposed. Yurochkin et al.^8^ proposed a Bayesian framework for FL. Claici et al.^9^ used KL divergence to fuse different models. Much work has also been done to improve the robustness of FL algorithms. Pillutla et al.^10^ proposed a robust and secure aggregation oracle based on the geometric median using a constant number of calls to a regular non-robust secure average oracle. Andrychowicz et al.^11^ proposed a meta-learning approach to coordinate the learning process in client/server distributed systems by using a recurrent neural network in the central server to learn how to optimally aggregate the gradients from the client models. Li et al.^12^ proposed a new framework for robust FL where the central server learns to detect and remove malicious updates using a spectral anomaly detection model, leading to targeted defense. Most of the algorithms cannot be directly compared or benchmarked as they address different problems in FL such as heterogeneity, privacy, adversarial robustness, etc. FedAvg is most commonly used because of its scalability to large datasets and comparable performance to other FL algorithms.

### Federated learning in histopathology

FL is especially important for histopathology departments, as it facilitates collaboration among institutions without sharing private patient data. One prominent challenge when applying FL to medical images, and specifically histopathology, is the problem of *domain adaptation*. Since most hospitals have diverse imaging methods and devices, images from a group of hospitals will be markedly different, and machine learning methods risk overfitting to non-semantic differences between them. Models trained using FL can suffer from serious performance drops when applied to images from previously unseen hospitals. Several recent works have explored applications of FL in histopathology, and grapple with this problem. Lu et al.^13^ demonstrated the feasibility and effectiveness of FL for a large-scale computational pathology studies. FedDG proposed by Liu et al.^14^ is a privacy-preserving solution to learn a generalizable FL model through an effective continuous frequency space interpolation mechanism across clients. Sharing frequency domain information enables the separation of semantic information from noise in the original images. Li et al.^15^ tackles the problem of domain adaptation with a physics-driven generative approach to disentangle the information about model and geometry from the imaging sensor^6^.
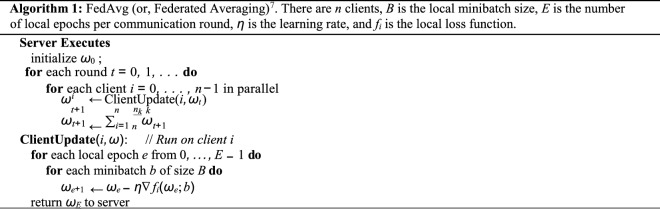


### Differential privacy

While FL attempts to provide privacy by keeping private data on client devices, it does not provide a meaningful privacy guarantee. Updated model parameters are still sent from the clients to a centralized server, and these can contain private information^16^, such that even individual data points can be reconstructed^17^. *Differential privacy* (DP) is a formal framework for quantifying the privacy that a protocol provides^18^. The core idea of DP is that privacy should be viewed as a resource, something that is used up as information is extracted from a dataset. The goal of private data analysis is to extract as much useful information as possible while consuming the least privacy. To formalize this concept, consider a *database D*, which is simply a set of datapoints, and a probabilistic function *M* acting on databases, called a *mechanism*. The mechanism is said to be (*ε, δ*)-*differentially private* if for all subsets of possible outputs $S \subseteq {\text{Range}}(M)$, and for all pairs of databases *D* and *D′* that differ by one element,

2 $$\Pr [M(D) \in S] \le \exp (\varepsilon )\Pr [M(D^{\prime}) \in S] + \delta .$$

When both *ε* and *δ* are small positive numbers, Eq. (2) implies that the outcomes of *M* will be almost unchanged in distribution if one datapoint is changed in the database. In other words, adding one patient’s data to a differentially private study will not affect the outcomes, with high probability.

The advantage of DP is that it is quantitative. It yields a numerical guarantee on the amount of privacy that can be expected, in the stochastic sense, where lower *ε* and *δ* implies that the mechanism preserves more privacy. The framework also satisfies several useful properties. When multiple DP-mechanisms are composed, the total operation is also a DP-mechanism with well defined *ε* and *δ*^19^. Also, once the results of a DP-mechanism are known, no amount of post-processing can change the (*ε, δ*) guarantee^20^. Hence, while FL alone does not guarantee privacy, we can apply FL in conjunction with DP to give rigorous bounds on the amount of privacy afforded to clients and patients who participate in the collaboration.

The simplest way to create a DP-mechanism is by adding Gaussian noise to the outcomes of a deterministic function with bounded sensitivity^21^. This method can be used in the context of training a machine learning model by clipping the norm of gradients to bound them, then adding noise, a process called *differentially private stochastic gradient descent* (DP-SGD)^22^. McMahan et al.^23^ applied this at scale to FL.

### Differential privacy for medical imaging

Past works have noted the potential solution DP provides for machine learning in the healthcare domain. Kaissis et al.^1^ surveyed privacy-preservation techniques to be used in conjunction with machine learning, which were then implemented for classifying chest X-rays and segmenting CT scans^24,25^. In histopathology, Lu et al.^13^ reported DP guarantees for a neural network classifier trained with FL, following Li et al.^26^. Their treatment involved adding Gaussian noise to trained model weights, however, neural networks weights do not have bounded sensitivity making their DP guarantee vacuous. A meaningful guarantee would require clipping the model weights before adding noise. We propose the more standard approach of DP-SGD, which clips gradient updates and adds noise, for use in histopathology.

### Multiple instance learning (MIL)

MIL is a type of supervised learning approach which uses a set of instances known as a bag. Instead of individual instances having an associated label, only the bag as a whole has one^27^. MIL is thus a natural candidate for learning to classify WSIs which must be broken into smaller representations due to size limitations. Permutation invariant operators for MIL were introduced by Tomczak et al.^28^ and successfully applied to digital pathology images. Isle et al.^29^ used MIL for digital pathology and introduced a different variety of MIL pooling functions, while Sudarshan et al.^30^ used MIL for histopathological breast cancer image classification. Graph neural networks (GNNs) have been used for MIL applications because of their permutation invariant characteristics. Tu et al.^31^ showed that GNNs can be used for MIL, where each instance acts as a node in a graph. Adnan et al.^32^ demonstrated an application of graph convolution neural networks to MIL in digital pathology and achieved state of the art accuracy on a lung sub cancer classification task.

## Method

**Figure 1:**
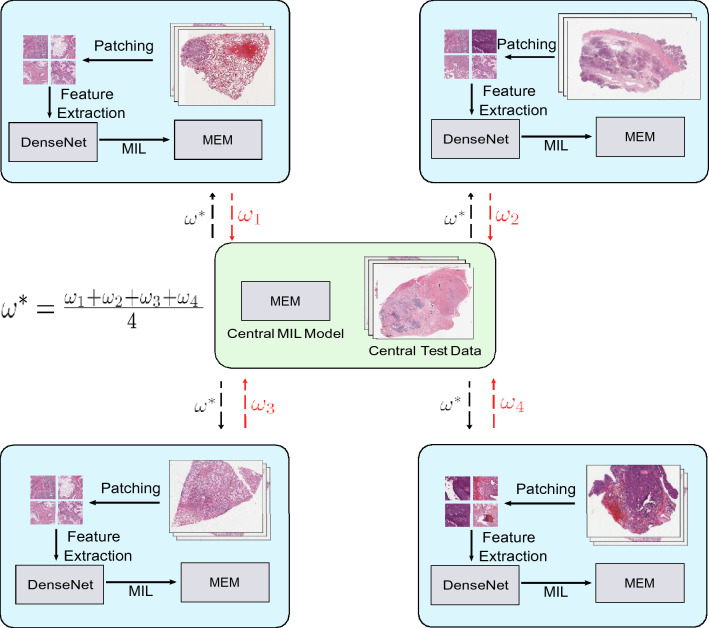
The proposed federated learning algorithm to train a MEM model^33^ for WSIs (disease) classification among multiple hospitals. Each client in FL is represented by a blue rectangle. Each client, first transforms their local WSIs into mosaics (sets of representative patches). The patches in each mosaic are converted to feature vectors using a DenseNet model^34^. Finally the sets of feature vectors are classified using a MEM model. A shared central MEM model is trained using FedAvg^6^ among multiple clients (mimicking hospitals). Furthermore, DP-SGD^22^ is used for training the central MEM model with strict privacy bounds.

Our proposed method (*local* to each client) consists of two steps, *bag preparation* and *Multiple-Instance Learning (MIL)*. In the first step, we extract multiple patches from the full-resolution WSI and create a mosaic (set) of patches. In the second step, we formulate the representation learning of WSIs as a set learning problem by applying a MEM model, an attention based MIL algorithm proposed by Kalra et al.^33^. The MEM model is locally trained through DP-SGD to provide quantitative privacy bounds, and the local MEM models are centrally aggregated through FedAvg. In this section, we discuss the bag preparation step and MIL. An overview of the proposed method is visualized in Fig. 1.

### Bag preparation

**Figure 2:**
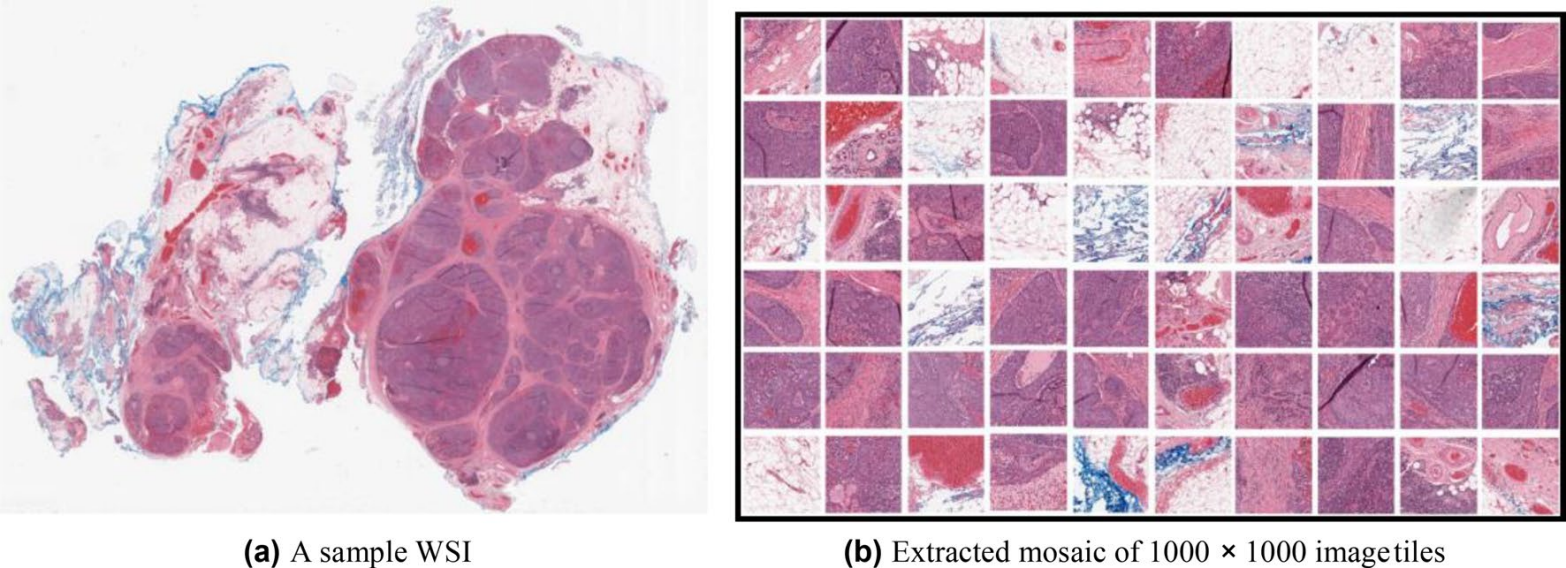
Illustration of a sample WSI and its mosaic extracted using the approach in Kalra et al.^35^.

A patch selection method proposed by Kalra et al.^35^ is used to extract representative patches (called *mosaics*) from each WSI. A sample WSI and its mosaic is illustrated in Fig. 2. The steps involved in creations of a mosaic are: (1) removal of non-tissue regions using colour thresholding; (2) grouping the remaining tissue-containing patches into a pre-set number of categories through a clustering algorithm; and (3) randomly selecting a portion of all clustered patches (e.g., 10%) within each cluster, yielding a *mosaic*. The mosaic is transformed into a bag *X* = *x*_1_*, ..., x*_*n*_ for MIL, where *x*_*i*_ is the feature vector of the *i*th patch, obtained through a pre-trained feature extractor network. We use a DenseNet model for the feature extractor^34^. Each patch in the mosaic has size 1000 × 1000 pixels at 20× magnification (0.5 mpp resolution).

### MIL method

**Figure 3:**
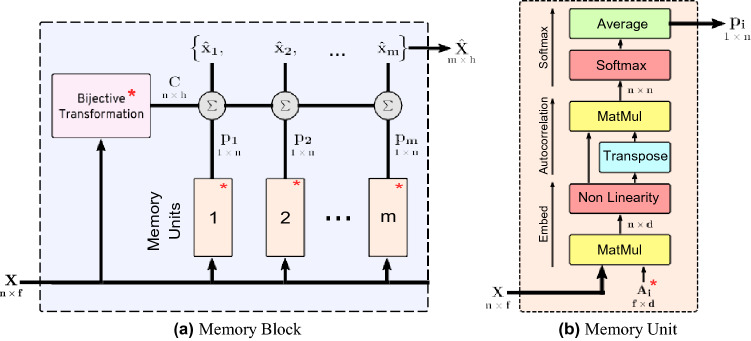
Schematic of a MEM model used for the classification of WSI mosaics. *X* is an input sequence containing a number *n* of *f* -dimensional vectors. (**a**) The memory block is a sequence-to-sequence model that takes *X* and returns another sequence $\hat{X}$. The output $\hat{X}$ is a permutation-invariant representation of *X*. A bijective transformation model (an autoencoder) converts the input *X* to a permutation-equivariant sequence *C*. The weighted sum of *C* is computed over different probability distributions *p*_*i*_ from memory units. The hyper-parameters of a memory block are (1) the dimensions of the bijective transformation *h*, and (2) the number of memory units *m*. (**b**) The memory unit has *A*_*i*_, a trainable embedding matrix that transforms elements of *X* to a *d*-dimensional space (memories). The output *p*_*i*_ is a probability distribution over the input *X*, also known as attention. The memory unit has a single hyper-parameter *d*, i.e. the dimension of the embedding space^33^ (* represents learnable parameters).

We used the MEM model proposed by Kalra et al.^33^ to get a single vector representation of all feature vectors of patches in a mosaic. MEM consists of memory units composed within a memory block. A *memory block* is the main component of MEM and produces a permutation invariant representation from a input sequence. Multiple memory blocks can be stacked together for modeling complex relationships and dependencies in set data. The memory block is made of memory units and a bijective transformation unit shown in Fig. 3. A memory unit transforms an input sequence into an attention vector. A higher attention value represents a higher “importance” of the corresponding element of the input sequence. Essentially, it captures the relationships among different elements of the input. Multiple memory units enable the memory block to capture many complex dependencies and relationships among the elements. Each memory unit consists of an embedding matrix **A**_**i**_ that transforms a *f*-dimensional input vector *x *_*j*_ to a *d*-dimensional memory vector *u*_*i j*_ according to

3 $$u_{ij} = \rho (x_{j} {\mathbf{A}}_{{\mathbf{i}}} ),$$

where *ρ* is some non-linearity. The memory vectors are stacked to form a matrix **U**_**i**_ = [*u*_*i*0_, ..., *u*_*in*_] of shape (*n d*). The relative degree of correlations among the memory vectors are computed using cross-correlation followed by a column-wise softmax and then taking a row-wise average,

4 $$\begin{aligned} S_{i} &= {\text{column-wise-softmax}}\left( {{\mathbf{U}}_{{\mathbf{i}}} {\mathbf{U}}}_{{\mathbf{i}}} ^{{\mathbf{T}}}\right), \\ p_{i} &= {\text{row-wise-average}}(S_{i} ). \\ \end{aligned}$$

The *p*_*i*_ is the final output vector (1 *n*) from the *i*th memory unit **U**_**i**_, as shown in Fig. 3. The purpose of each memory unit is to embed feature vectors into another space that could correspond to a distinct “attribute” or “characteristic” of instances. The cross correlation of the calculated attention vectors highlights instances which are highly suggestive of those attributes. Memory vectors are non-normalized as the magnitude may play an important role during the cross correlation.

In summary, a memory block is a sequence-to-sequence model, i.e., it transforms a given input sequence *X* = *x*_1_, ..., *x*_*n*_ to another representative sequence $\hat{X} = \widehat{{x_{1} }}, \ldots \widehat{{x_{m} }}$. A memory block contains *m* memory units, each of which takes sequential data as an input and generates an attention vector. These attention vectors are subsequently used to compute the final output sequence. By design, the output sequence is invariant to element-wise permutations of the input sequence as needed for MIL.

## Experiments and discussion

We validated the performance of FL for the classification of histopathology images using a simulated distributed environment and also using real-world hospital data. Previous studies have mostly experimented with a fixed number of clients having similar distributions of data^1,13,36^. Since real-world data is not necessarily IID, it is important to study the effect of non-IID data on the performance of FL, specifically *FedAvg*. Furthermore, we provide a privacy analysis of the method through the differential privacy framework, suggesting that FL can outperform non-collaborative training while maintaining a strong privacy guarantee.

In the *first experiment series*, we vary the number of clients, with each client representing one hospital. To make our simulated environment better approach the non-IID real-world data, each client can have a different number of patients and a different distribution of cancer sub-types. In the *second experiment series*, we calculate the privacy bound of differentially private FL using real-world hospital data. We used the available attributes in TCGA to divide the dataset across the tissue origin site (hospital) and created four client datasets as shown in Table 2.

### Lung cancer dataset—LUAD vs LUSC classification

Lung Adenocarcinoma (LUAD) and Lung Squamous Cell Carcinoma (LUSC) are two main sub-types of non-small cell lung cancer (NSCLC) that account for 65–70% of all lung cancers^37^. An automated classification of these two main sub-types of NSCLC is a crucial step to assist pathologists for more informed diagnoses^37,38^. We obtained 2580 hematoxylin and eosin (H&E) stained WSIs of lung cancer from TCGA^39^, comprising about two TB of data. The images were split into two groups of 1806 training, and 774 testing samples WSIs^33^. We transformed each raw image into a mosaic^35^, and then into a bag of features *X* using a pre-trained DenseNet^34^. From the data, we carried out two experiment series by varying the parameters of FedAvg, or by varying the data distributions across clients. These experiment series are discussed as follows.

### Experiment series 1—effect of number of clients and data distributions

**Figure 4:**
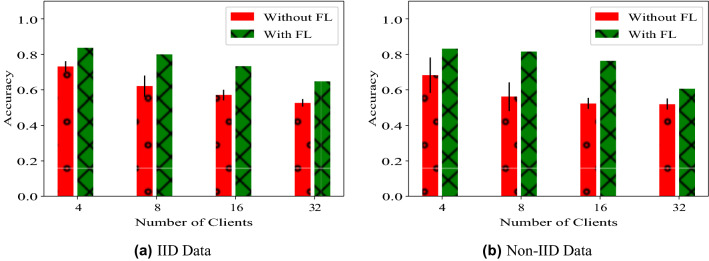
Comparison of the mean accuracy across clients versus the accuracy of the central model trained with FL for the fabricated clients (not the real hospitals). The accuracy is computed on two types of data distribution settings across clients—IID and Non-IID.

**Figure 5:**
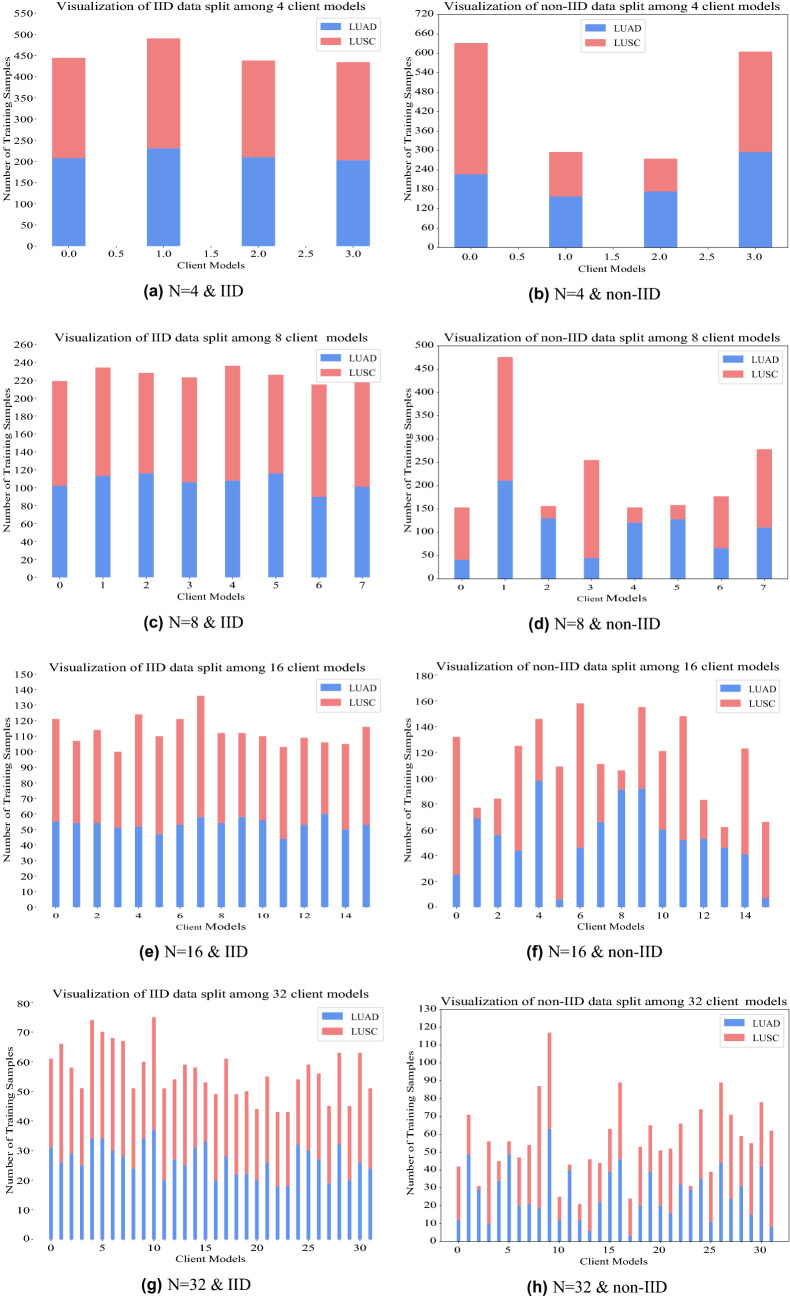
Visualisation of IID and non-IID distribution of data among client models.

We studied the effect of IID and non-IID distributions on the performance of FedAvg by randomly dividing the training images without replacement among different clients (hospitals). We also varied the number of clients (*n*) while keeping the total number of images fixed. IID data is generated by uniformly dividing each cancer sub-type, i.e. LUAD and LUSC, among different clients. For each cancer sub-type, a probability distribution is created by assigning a random value to each client and then dividing it by the total sum. Subsequently, images are divided among different clients by sampling from the probability distribution. FL achieves superior performance for both IID and non-IID distributions of data compared to non-collaborative training. FL performs comparably to centralized training for reasonably sized datasets (*n* = 4, 8). Results are summarized in Table 1 and Fig. 4. The number of training samples for each client model is in Fig. 5.

**Table 1:** Evaluation on different data distributions.

Data distribution	Number of clients *n*	Accuracy		
		Without FL	With FL	Centralized
IID	4	0*.*731 ± 0*.*03	0*.*824 ± 0*.*02	0*.*848 ± 0*.*02
	8	0*.*620 ± 0*.*06	0*.*780 ± 0*.*05	
	16	0*.*570 ± 0*.*03	0*.*726 ± 0*.*06	
	32	0*.*527 ± 0*.*02	0*.*641 ± 0*.*09	
Non IID	4	0*.*682 ± 0*.*10	0*.*824 ± 0*.*01	0*.*848 ± 0*.*02
	8	0*.*561 ± 0*.*08	0*.*823 ± 0*.*05	
	16	0*.*524 ± 0*.*03	0*.*750 ± 0*.*06	
	32	0*.*520 ± 0*.*03	0*.*550 ± 0*.*20	

Centralized accuracy denotes the accuracy when the data is centralized. The accuracy without FL is the mean and standard deviation of accuracy values across multiple clients without any collaboration. The accuracy with FL is the mean and standard deviation of the central model trained at the end of FL evaluated on each client dataset.

We compared the performance with and without FedAvg for each setting. In total we tested 16 experimental settings in Table 1. In each of the experiments, the server model trained using FedAvg outperformed the models trained using local client datasets, showing the advantage of collaboration. As the total dataset is divided into smaller partitions for more clients, both client and server model performances deteriorate. We used SGD optimizer with learning rate = 0*.*01. The local epoch for each client was set to 1 and the server model was trained for 250 communication rounds. We visualize the relative improvement of FedAvg in Table 1.

### Experiment series 2—real-world setting

In the second experiment series, we considered the effect of distributional differences from different source hospitals, and a requirement to preserve privacy. Histopathology images can differ greatly, among others depending on the staining and imaging protocols of the source hospital. We selected seven hospitals from the TCGA dataset, four to act as clients in FL, and an additional three to provide externally collected data for model robustness testing. The distribution of images by hospital is described in Table 2. For each of the four clients, we divided their available images in an 80:20 ratio for training and internal testing datasets, respectively. Then we combined the images from the remaining three hospitals into a single external validation dataset to study the effects of distributions shifts on FedAvg.

**Table 2:** Source hospitals for test/train and external dataset and their data distribution.

Dataset type	Source hospital (clients)	LUAD images	LUSC images	Total
Train/test	International Genomics Consortium	189	78	267
	Indivumed	94	117	211
	Asterand	90	117	207
	Johns Hopkins	121	78	199
External	Christiana Healthcare	169	54	223
	Roswell Park	35	75	110
	Princess Margaret Hospital (Canada)	0	52	52

In this experiment, we use Differential Private Federated Learning (DP-FL) to ensure data privacy. Differential Privacy (DP) was not considered in experiment series 1 since the objective was to study the effects of data size, distribution, and the number of clients on the performance of distributed learning/federated learning in general. In experiment series 2, we compared the performance of privacy-preserving FL training with both centralized training and non-collaborative training. In the FL training, the four hospitals act as clients collaborating to train one central model. Performance is evaluated on each client’s internal test set, as well as the external validation set. For comparison, we train a single model on the combined (centralized) training datasets which gives an upper bound on what could be achieved in the absence of privacy regulations. Finally, in the non-collaborative setting each client hospital trains their own model on only their own training dataset. We used DP-SGD to train the FL and combined models and computed the privacy guarantees (*ε*, *δ*) using a Rényi DP accountant^4^. It was observed that the MEM model was sensitive to DP-SGD hyper parameters. We used a vectorized Adam optimizer^40^ with the following hyper-parameter values^22^: epochs = 180, training set size = 705, batch size = 32, gradient clipping norm = 1*.*0, Gaussian noise standard deviation = 4*.*0, number of microbatches = 32, learning rate = 2 × 10^−5^. Ablation study is provided in the Table 3.

**Table 3:** Ablation study of DP hyperparameters (gradient clipping and noise multiplier).

Gradient clipping	Noise multiplier	Privacy budget (*ε*)	Test accuracy	External accuracy
1.0	4	2.90	0.815	0.740
1.5	4	3.26	0.759	0.719
2.0	4	3.89	0.765	0.732
1.0	6.0	2.34	0.832	0.737
1.0	2.0	10.01	0.782	0.748

As shown in Table 4, FL training achieves strong privacy bounds (*ε* = 2*.*90 at *δ* = 0*.*0001) with better performance than non-collaborative training, comparable to centralized training. This demonstrates that FL could be effectively used in clinical settings to ensure data privacy with no significant degradation in performance. Results are shown in Table 4. FedAvg achieves comparable performance to centralized training without explicitly sharing private data with strong privacy guarantees. Due to distribution shifts, accuracy decreases on external validation for both Federated Learning and centralized training. Therefore, we experimentally demonstrate the Federated Learning can be used for medical image analysis in real-world setting without explicitly sharing data, while achieving similar performance to centralized training with data sharing.

**Table 4:** Evaluation of collaborative and non-collaborative learning on Test and External Datasets using DP-SGD, achieving privacy parameter *ε* = 2*.*90 for *δ* = 0*.*0001.

Source hospital	Non-collaborative training		DP-FL training		FL training		Combined training	
	Test	External	Test	External	Test	External	Test	External
International Genomics Consortium	0.654	0.631	0.823 ± 0.01	0.707 ± 0.01	0.823 ± 0.01	0.741 ± 0.01	0.839 ± 0.01	0.768 ± 0.003
Indivumed	0.648	0.556						
Asterand	0.709	0.701						
John Hopkins	0.681	0.600						

For FL and combined training we report the mean accuracy and standard deviation across the client’s test datasets. On the external dataset we ran the experiments using three random initializations, and report the mean accuracy and standard deviation across them.

## Conclusions

There is a vast reserve of knowledge in mass archives of clinical data held by hospitals which remains mostly untapped due to many confidentiality and privacy concerns. In this work, we proposed *differentially private federated learning* as a potential method for learning from decentralized medical data such as histopathology images. Federated learning allows training models without explicitly sharing patient data and thus mitigates some confidentiality and privacy issues associated with clinical data. Differential privacy supplements this with quantitative bounds on the amount of privacy provided. We demonstrated the efficacy of federated learning (FedAvg) with simulated real-world data, using both IID and non-IID data distributions. Private federated learning achieves a comparable result compared to conventional centralized training, and hence it could be considered for distributed training on medical data.

## Data Availability

The publicly available dataset of 30,072 WSIs from TCGA^39^ (Genomic Data Commons GDC) was used for conducting this study.
